# Comprehensive analysis to identify PUS7 as a prognostic biomarker from pan-cancer analysis to osteosarcoma validation

**DOI:** 10.18632/aging.205863

**Published:** 2024-05-30

**Authors:** Baokang Dong, Binqi Wang, Meng Fan, Jingyu Zhang, Ziqin Zhao

**Affiliations:** 1 Department of Orthopaedics, Tianjin First Central Hospital, Nankai University, Tianjin 300192, China; 2 Department of Bone Tumor and Soft Tissue Oncology, Tianjin Hospital of Tianjin University, Tianjin 300211, China; 3 Department of Pathology, Tianjin Hospital of Tianjin University, Tianjin 300211, China

**Keywords:** pan-cancer, PUS7, cell cycle, osteosarcoma, proliferation

## Abstract

Aim: Pseudouridylation has demonstrated the potential to control the development of numerous malignancies. PUS7(Pseudouridine Synthase 7) is one of the pseudouridine synthases, but the literature on this enzyme is limited to several cancer types. Currently, no investigation has been performed on the systematic pan-cancer analysis concerning PUS7 role in cancer diagnosis and prognosis.

Methods: Employing public databases, including The Cancer Genome Atlas (TCGA), Genotype-Tissue Expression Project (GTEx), Human Protein Atlas (HPA), UALCAN and Tumor Immune Single-cell Hub (TISCH), this work investigated the PUS7 carcinogenesis in pan-cancer. Differential expression analysis, prognostic survival analysis and biological function were systematically performed. Furthermore, PUS7 potential as an osteosarcoma biomarker for diagnosis and prognosis was assessed in this study.

Results: The findings indicated that PUS7 was overexpressed in the majority of malignancies. High PUS7 expression contributed to the poor prognosis among 11 cancer types, including Adrenocortical Cancer (ACC), Bladder Cancer (BLCA), Liver Cancer (LIHC), Kidney Papillary Cell Carcinoma (KIRP), Mesothelioma (MESO), Lower Grade Glioma (LGG), Kidney Chromophobe (KICH), Sarcoma (SARC), osteosarcoma (OS), Pancreatic Cancer (PAAD), and Thyroid Cancer (THCA). In addition, elevated PUS7 expression was linked to advanced TNM across multiple malignancies, including ACC, BLCA, KIRP, LIHC and PAAD. The function enrichment analysis revealed that PUS7 participates in E2F targets, G2M checkpoint, ribosome biogenesis, and rRNA metabolic process. Moreover, PUS7 is also a reliable biomarker and a potential therapeutic target for osteosarcoma.

Conclusions: In summary, PUS7 is a putative pan-cancer biomarker that reliably forecasts cancer patients’ prognosis. In addition, this enzyme regulates the cell cycle, ribosome biogenesis, and rRNA metabolism. Most importantly, PUS7 possibly regulates osteosarcoma initiation and progression.

## INTRODUCTION

Being the primary cause of death worldwide, the burden of cancer morbidity and mortality is expanding dramatically [1]. In 2020, 9,958,133 casualties were reported worldwide, with 19,292,789 new cases [2]. Surgery remains the preferred treatment option, but the variety of cancer therapies has increased throughout the years. For instance, precision oncology is quickly reshaping cancer treatment guidelines as genomic analysis is widely employed for diagnostic and therapeutic purposes in multiple tumors. Furthermore, a variety of small-molecule drugs have exhibited impressive clinical efficacy. Osteosarcoma is an extensive and aggressive tumor compared to malignancies of epithelial origin. Individuals with metastatic or recurring osteosarcoma have an overall survival rate of approximately 25% [3, 4]. Surgery and adjuvant chemotherapy are the mainstays of osteosarcoma treatment and have remained constant over the past 30 years due to its extensive malignancy. Therefore, researchers continue their search for novel treatments for this disease. Several targeted drugs for osteosarcoma have proceeded to clinical trials with tremendous therapeutic effects [5]. Thus, the future of osteosarcoma treatment may lie in the application of small-molecule therapeutic drugs in conjunction with operations and chemotherapy. The advancement of high-throughput sequencing has boosted the discovery of new drug targets. Tumors are highly heterogeneous but with a certain level of homogeneity; hence, some oncogenes are found in multiple cancer types. For instance, TP53 is a key tumor suppressor gene mutated in more than half of human cancers [6]. Pan-cancer analysis has enabled researchers to identify biomarkers involved in various malignancies using sequencing data [7], which are promising therapeutic targets.

Pseudouridine(Ψ), an isomer of uridine, is the most abundant and widespread epigenetically-modified RNA in organisms [8]. Despite that, the biological role of pseudouridine is not fully understood in cancer. Pseudouridine synthases (PUSs) catalyze pseudouridine formation, which classified into six families: TruA, TruB, TruD, RsuA, RluA, and PUS10 [9]. Emerging studies have identified that PUSs are associated with tumorigenesis and cancer progression. As for instance, by directly triggering the transcription of HIF-1, the elevated PSU7 expression in CRC (colorectal cancer) tissues could control angiogenesis and metastasis [10]. PUS7 belongs to the TruD class. The expression of PUS7 and its catalytic activity are necessary for the development of glioblastoma stem cells (GSC) tumorigenesis, and PUS7 pharmacological inhibitors prevent the growth of tumors and extend the lifespan of tumor-bearing mice [11]. Additionally, PUS7 promotes CRC cell growth via effectively stabilizing SIRT1 to stimulate Wnt/-catenin pathway [12]. Du et al. also discovered PUS7 overexpression accelerates colon cancer cell proliferation and invasion via PI3K/AKT/mTOR Signaling Pathway [13]. PUS7 has been proven to be a valid biomarker for lung cancer diagnosis in recent research [14]. Nonetheless, systematic research on PUS7 function in various malignancies remains lacking.

This study identified the aberrant expression of PUS7 in tumor and normal tissues and confirmed the predictive value in cancer patient prognosis. In addition, PUS7 regulates cell division and cell cycle, as well as ribosome biosynthesis and rRNA metabolism. Finally, we identified the PUS7’s oncogene role in osteosarcoma. In conclusion, PUS7 is a novel and effective biomarker, thus, an attractive molecule target for cancer treatment.

## MATERIALS AND METHODS

### Data collection and expression analysis of PUS7

The mRNA expression matrix was downloaded from the Cancer Genome Atlas (TCGA) database (https://portal.gdc.cancer.gov/) across 33 cancer types. The bulk mRNA sequencing data of osteosarcoma (OS) in TCGA-TARGET and GSE21257 was obtained [15]. Of course, we downloaded the relevant clinical information, including OS, PFS, DFI, DSS, and clinical features. Then, using the TCGA and Genotype-Tissue Expression Project (GTEx) datasets, the differential expression value of PUS7 between normal and malignant samples across 33 cancer types were examined. Finally, the UALCAN (https://ualcan.path.uab.edu/index.html) web platform was utilized to ascertain PUS7’s protein level.

### Immunohistochemistry

The Human Protein Atlas (HPA) (https://www.proteinatlas.org/) provided the immunohistochemistry images of PUS7 protein expression in 15 different cancer types and corresponding normal tissues. Meanwhile,10 pairs of paraffin-embedded osteosarcoma and adjacent samples were taken from the Tianjin Hospital of Tianjin University and none of them received preoperative chemotherapy. More crucially, all patients have approved the use of the surgical material for academic research and publications. All methods were approved by The Institutional Review Committee and the Medical Ethics Committee of the Tianjin Hospital of Tianjin University. The slides were incubated with anti-PUS7 (1:1000; ab289857, Abcam, Rabbit), following the manufacturer’s protocol. Two pathologists independently investigated and quantified the slide images. The IHC intensity score is 0 (negative), 1 (weak brown), 2 (medium brown), or 3 (strong brown). The staining content was categorized into five levels: 0 (≤10%), 1 (11%-25%), 2 (26%-50%), 3 (51%-75%), or 4 (>75%). The staining value was established through the multiplication of intensity scores and extent scores.

### Relationship between PUS7 expression, prognosis, and clinical features

Four survival indicators (OS, DSS, DFI, and PFI) were utilized to examine the connection between PUS7 expression and cancer patients’ prognosis. The survival analysis was carried out via the survival R program. Meanwhile, the best-cutoff point was obtained through the “surv cutpoint” function in the survminer R package. Using the optimal cutoff point for PUS7 expression level, the patients were later divided into two groups for each cancer type and then modeled the Kaplan-Meier survival curves. Additionally, a univariate Cox regression analysis was conducted to ascertain the predictive significance of PUS7 expression. Finally, the association between PUS7’s expression value and clinical data was explored in this study.

### Functional enrichment analysis of PUS7

The PUS7 has been identified as an un-favor oncogene in 11 cancer types in our work. To investigate PUS7’s oncogenic role in malignant tumors, we extracted RNA sequences of the following cancer types, including Bladder Cancer (BLCA), Kidney Papillary Cell Carcinoma (KIRP), Lower Grade Glioma (LGG), Liver Cancer (LIHC), Sarcoma (SARC), Thyroid Cancer (THCA). For these cancer types, PUS7 exhibited a significant adverse effect. These cancer types also accounted for more than 200 individuals, which might increase the accuracy of the functional analysis result. Cases were classified into PUS7-high and -low subsets relied on PUS7’s median value in each cancer type. Enriched gene sets were identified using the gene set enrichment analysis (GSEA).

### PUS7-related regulatory gene enrichment analysis

Similarly, the RNA-seq matrix was extracted from BLCA, KIRP, LGG, LIHC, SARC, and THCA patient samples to identify PUS7-related regulatory genes. The patients were split into PUS7-high and PUS7-low subsets on the basis of PUS7 median value in each cancer type. Subsequently, a differential expression analysis between PUS7-high and PUS7-low group was performed to detect the differentially expressed genes (DEGs) (p < 0.05, Log FC > 1). The association between PUS7 and these DEGs was later determined using Spearman’s correlation analysis for each cancer type (p < 0.05, Cor > 0.4). PUS7-related regulatory genes were identified as the genes that intersected for these closely related genes. Then, using clusterProfiler R package [16], the gene enrichment analyses were performed on PUS7-related regulatory genes. Finally, the effector function of these regulatory genes was ascertained using the Metascape online platform (https://metascape.org/gp/index.html).

### Single-cell analysis

This study estimated the PUS7 expression level in cell types across numerous cancers via Tumor Immune Single-cell Hub (TISCH) database, an online platform designed for multiple single-cell analyses (http://tisch.comp-genomics.org/home/). Further research for PUS7 in single-cell resolutions was performed using the osteosarcoma GSE152048 dataset downloaded from GEO database [17]. First, a quality control step was performed to exclude unsuitable cells (RNA counts 200 - 7000; mitochondrial gene expression: < 5%). Data were normalized using the “LogNormalize” function with 10,000 scale factor. Meanwhile, the influence of UMIs and mitochondrial content (%) was eliminated using Seutat’s ScaleData function. Subsequently, the batch effect was removed using the harmony R package. The top 30 principal components and top 2000 variable genes were selected for cell clustering and the uniform manifold approximation and projection (UMAP) visualization [18]. Finally, Canonical marker genes identified in previous studies were employed to mark the cell type.

### Statistical analysis

Analyses between the two groups were performed using the Wilcoxon test, while the one-way analysis of variance (ANOVA) test was utilized for three or more groups. All statistical calculations were carried out with GraphPad and R studio.

### Data availability

The data involved in our work are available in the TCGA (https://portal.gdc.cancer.gov/) and GEO (https://www.ncbi.nlm.nih.gov/geo/).

## RESULTS

### PUS7 expression across cancers

**Figure 1:**
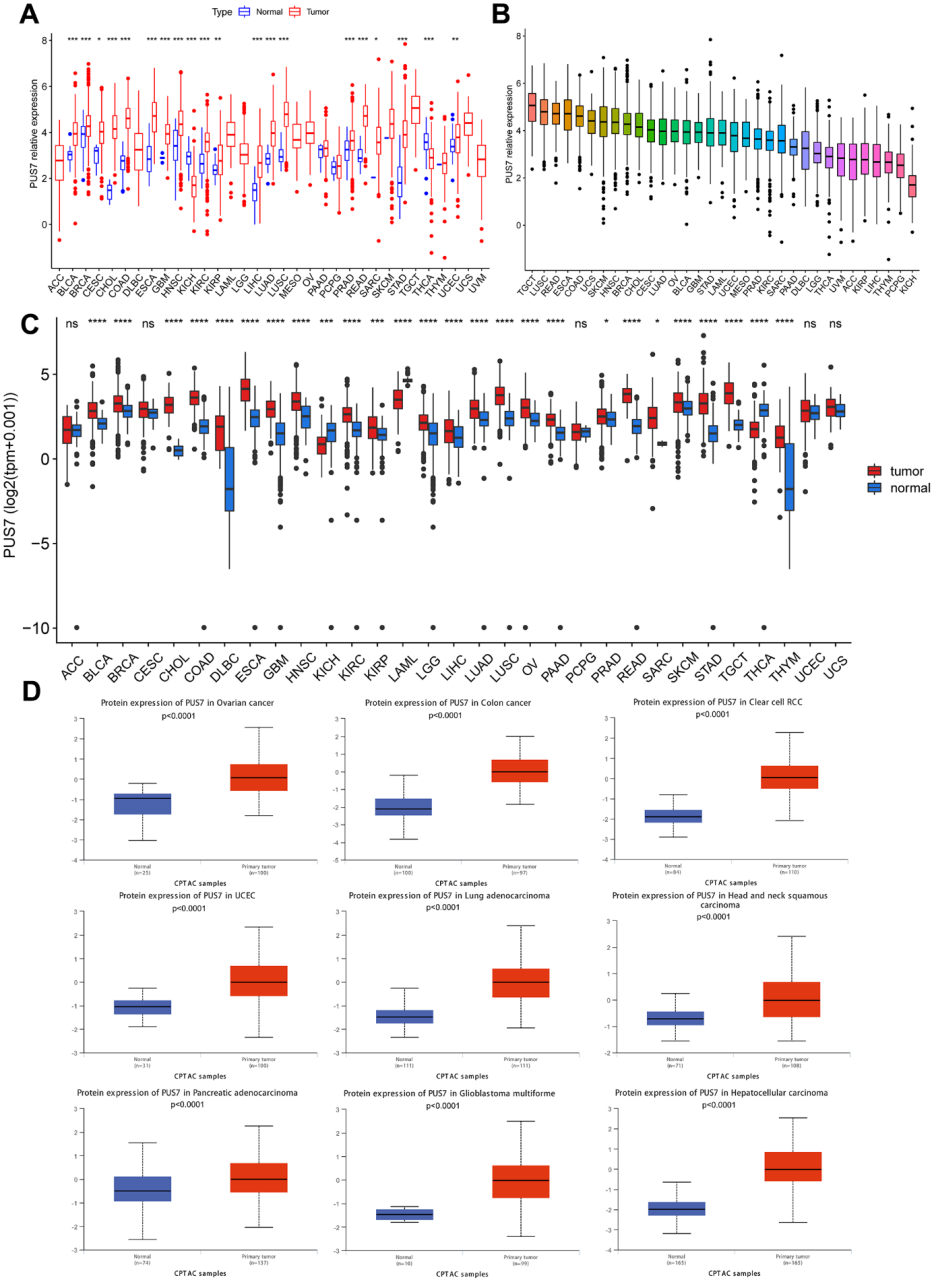
**Differential expression of PUS7 in pan-cancer.** (**A**) Comparison of PUS7 expression between tumor and normal samples in TCGA database. (**B**) Expression of PUS7 in tumor tissue for 33 cancer types. (**C**) Comparison of PUS7 expression between tumor and normal samples in TCGA and GTEx database. (**D**) Comparison of PUS7 protein level between tumor and normal samples.

**Figure 2:**
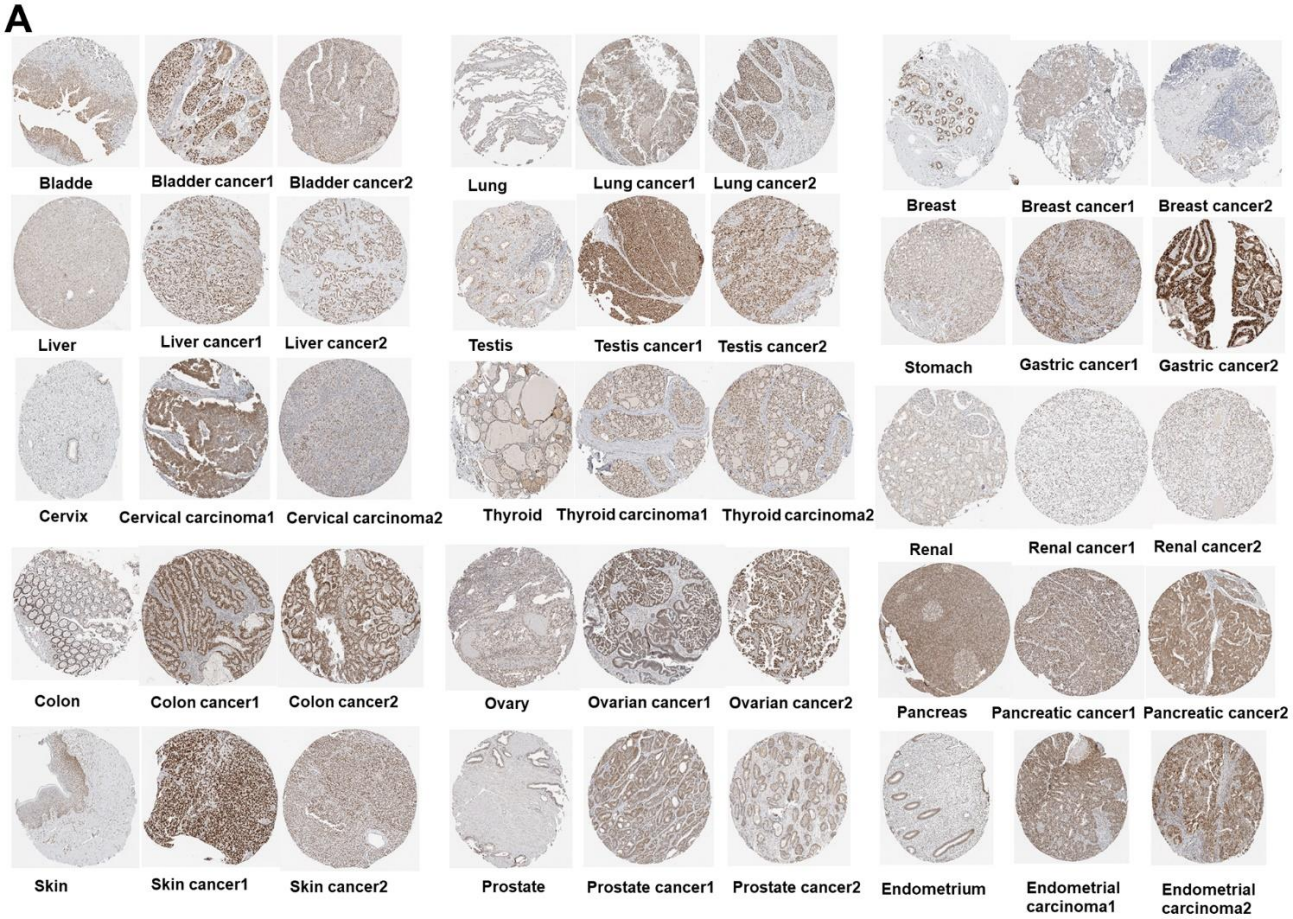
(**A**) Immunohistochemical images of the normal (left) and tumor (right) group with PUS7 protein expression.

The PUS7 expression in normal and tumor tissues was assessed using the TCGA database. In this study, PUS7 was significantly upregulated in most tumor tissues, including BLCA, Breast Cancer (BRCA), Cervical Cancer (CESC), Bile Duct Cancer (CHOL), Colon Cancer (COAD), Esophageal Cancer (ECSA), Glioblastoma (GBM), Head and Neck Cancer (HNSC), Kidney Clear Cell Carcinoma (KIRC), KIRP, LIHC, Lung Adenocarcinoma (LUAD), Lung Squamous Cell Carcinoma (LUSC), Prostate Cancer (PRAD), Rectal Cancer (READ), SARC, Stomach Cancer (STAD), and Endometrioid Cancer (UCEC). Conversely, Thyroid Cancer (THCA) and Kidney Chromophobe (KICH) tumor tissues showed a considerable decrease in PUS7 expression (Figure 1A). Additionally, PUS7’s relative expression value in different cancer tissues were examined. It was discovered that PUS7 expression was the highest in Testicular Cancer (TGCT), LUSC, and READ tissues and the lowest in KICH tissues (Figure 1B). Samples from TCGA and GTEx databases were merged to examine PUS7 expression in cancer and paraneoplastic tissues because the TCGA database has limited normal samples. It was found that 25 out of 33 cancer types had significantly higher levels of PUS7 in tumor tissues, contrary to KICH, THCA, and Acute Myeloid Leukemia (LAML). In summary, PUS7 expression was elevated in most cancers, suggesting its oncogene role in cancers. The PUS7 protein level in tumor and normal samples was further assessed using the UALCAN online platform, but the online proteomic data was limited. Consequently, this study identified a considerable increase in PUS7 protein levels in ovarian cancer, colon cancer, ccRCC, UCEC, LUAD, HNSC, Pancreatic Cancer (PAAD), LGG and LIHC tumor tissues, which was in line with the RNA-seq analysis (Figure 1D). The HPA database was also utilized to obtain immunohistochemical images. The protein level of PUS7 varied considerably in 15 tumor tissues (https://www.proteinatlas.org/ENSG00000091127-PUS7/pathology) and corresponding normal tissues (https://www.proteinatlas.org/ENSG00000091127-PUS7/tissue, version: 23.0) (see Figure 2A).

### Prognostic value of PUS7 for cancer patients

**Figure 3:**
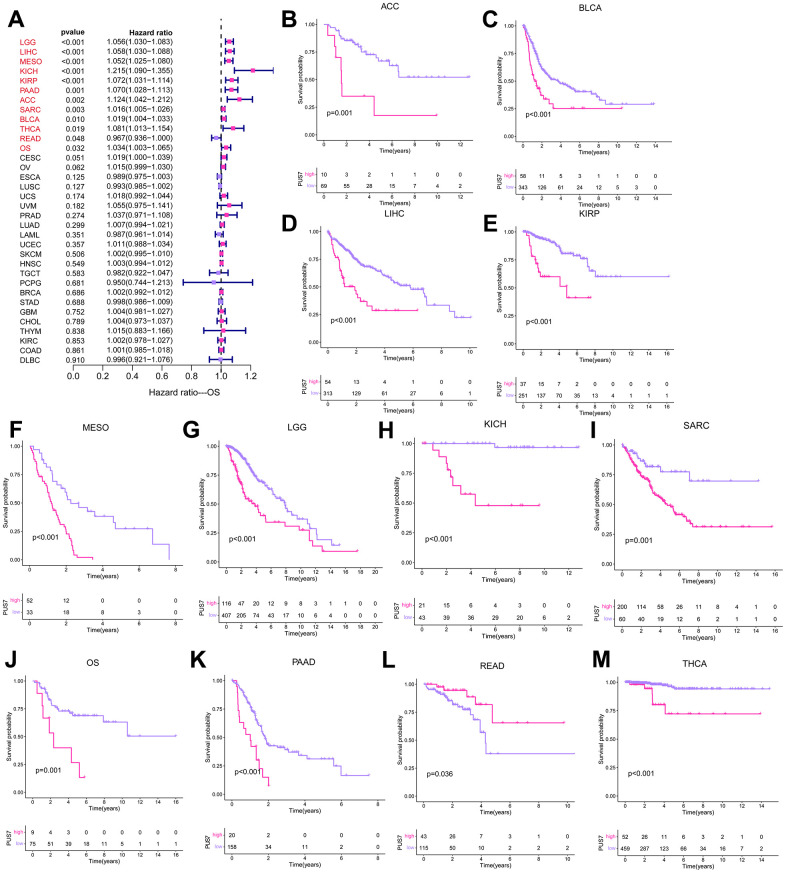
**PUS7 prognostic value across cancers.** (**A**) The forest plot exhibited the prognostic role of PUS7 in cancers obtained via the univariate Cox regression method. The cancer type in red indicates PUS7 as a risk factor with statistical significance. (**B**–**M**) Kaplan–Meier analysis of the association between PUS7 expression and OS.

**Figure 4:**
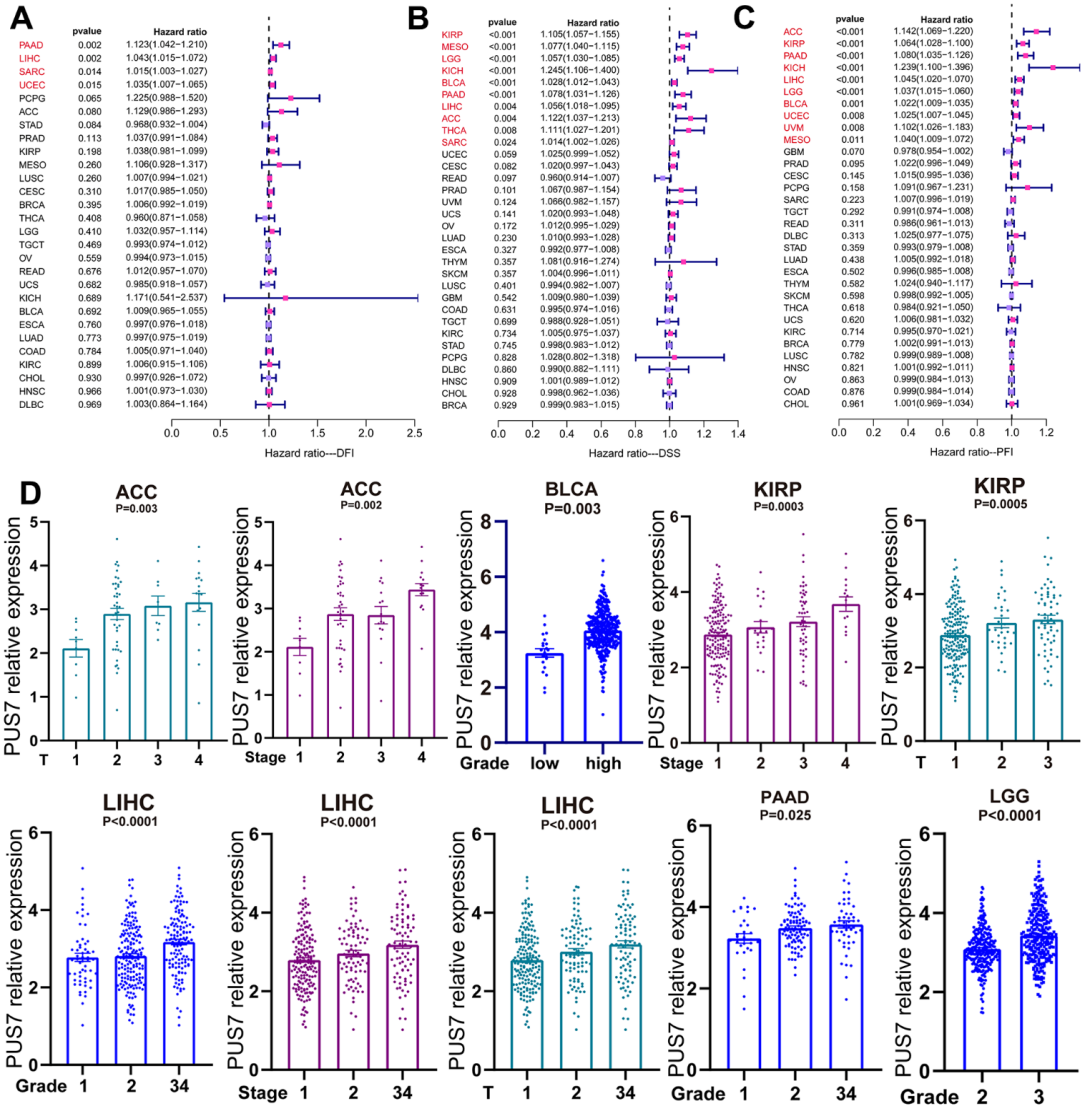
**PUS7 expression positively correlated with tumor progression.** (**A**) The forest plot exhibited the association between PUS7 expression and DFI (**A**), DSS (**B**), and PFI (**C**) in cancers. (**D**) Association between PUS7 expression and tumor stage, metastasis, grade, and size.

The potential of PUS7 as a prognostic biomarker was explored as PUS7 was overexpressed in most malignancies. Univariate regression and Kaplan-Meier survival analyses for each cancer type were implemented to examine the association between PUS7 expression and the cancer patients’ prognosis, concentrating on OS, DFI, DSS, and PFI. There exists a strong correlation between poor outcomes and upregulated PUS7 expression in 11 different types of cancer patients, including Adrenocortical Cancer (ACC), BLCA, LIHC, KIRP, Mesothelioma (MESO), LGG, KICH, SARC, OS, PAAD, and THCA, which indicated PUS7 was probably a proto-oncogene (Figure 3A–3M). High PUS7 expression was connected with shorter DFI among tumor patients with PAAD, LIHC, SARC, and UCEC (Figure 4A). Similarly, patients exhibiting higher PUS7 expression demonstrated poor DSS and PFI across numerous malignancies (see Figure 4B, 4C). Notably, PUS7 was identified as a significant risk factor for SARC in the OS, DSS, and DFI analysis, suggesting the critical function of this protein in SARC. The correlation between PUS7 expression and clinical features was evaluated to confirm the role of PUS7 in cancer progression. The clinical features reflect tumor progression to some extent, as observed in LIHC, where PUS7 expression positively correlated with tumor stage, size, and grade (Figure 4D). Furthermore, PUS7 possibly impacts other tumor progression, such as ACC, BLCA, and PAAD. In summary, the current analysis identified PUS7 as a potential promoter of tumor initiation and progression.

### Analysis of PUS7 in single cells

**Figure 5:**
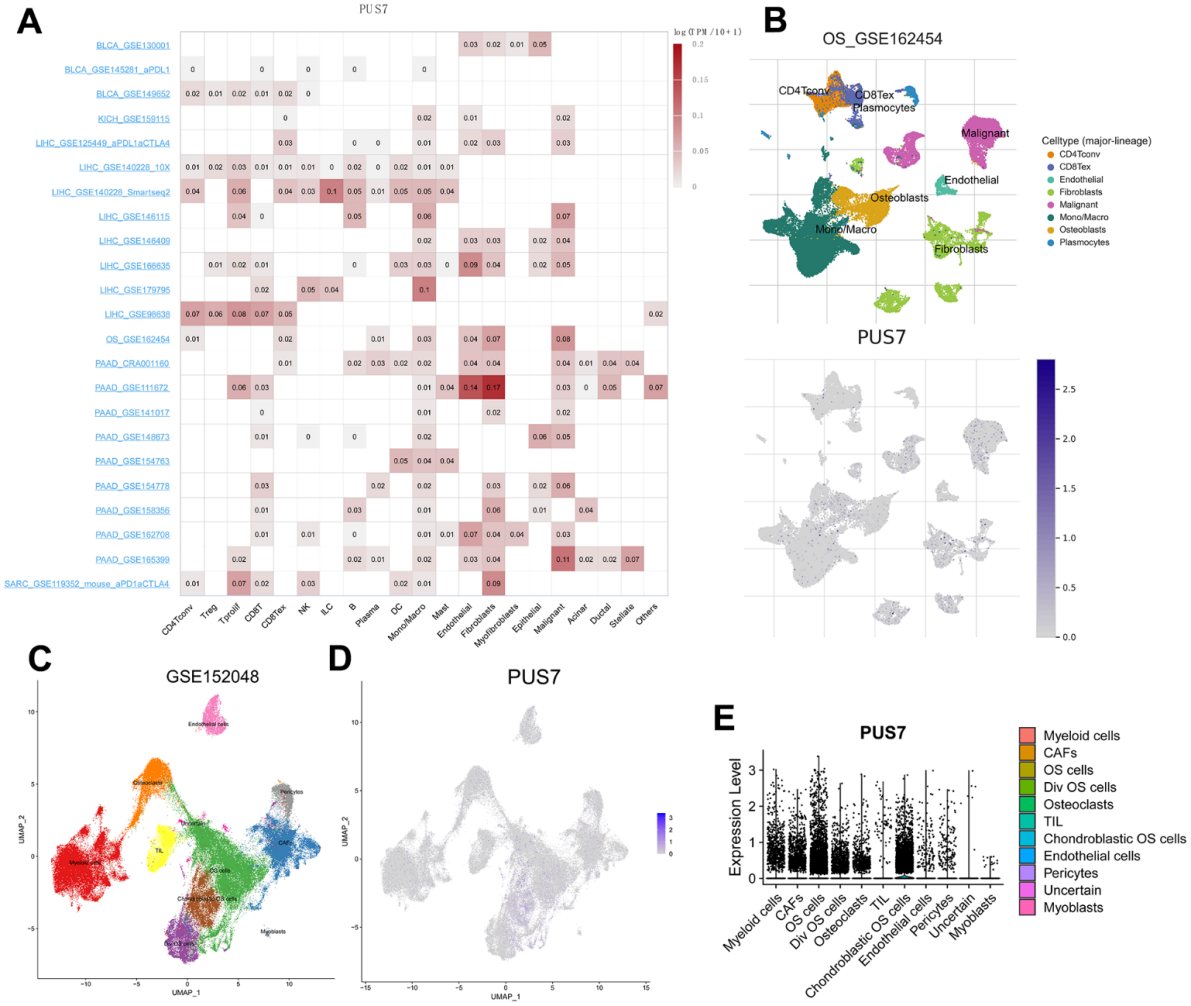
**Single-cell analysis of PUS7 in cancers.** (**A**) Summary of PUS7 expression of 23 cell types in 31 single-cell datasets. (**B**) UMAP plots of all single cells of osteosarcoma patients, showing all cell types in the plot. (**C**) UMAP plot of all cell clusters in the GSE150248 dataset. (**D**) UMAP plot and Violin plots. (**E**) showing the expression of PUS7 in each cell type.

Previous carcinoma research focused on tumor cells without recognizing the significance of non-cancerous cells. Scientists have recently begun to consider cancer an evolutionary ecosystem evolutionary ecosystem in which tumor microenvironment (TME) and tumor cells interact constantly and dynamically [19]. Various subpopulations of the same cell type may vary in distribution, number, and metabolic activity owing to TME’s great heterogeneity. Similarly, an oncogene can affect both tumor cells and other cells, thus, its role in tumor development is complex. Single-cell sequencing is a powerful tool to analyze oncogene expression at the single-cell resolution. We discovered PUS7 existed in malignant cells as well as in endothelial, fibroblast, and immune cells, including macrophages and T cells employing scRNA sequencing of BLCA, KICH, LIHC, SARC, and PAAD (Figure 5A). Focusing on the distribution of PUS7 in osteosarcoma, fibroblasts and cancer cells had the highest levels of PUS7 expression (Figure 5B). Subsequently, another osteosarcoma single-cell database—GSE150248, was utilized to validate the PUS7 expression in each cell type (Figure 5C). The findings exhibited that PUS7 was highly expressed in myeloid cells, cancer cells, and fibroblasts (Figure 5D, 5E). As PUS7 is highly expressed with fibroblasts and immune cells, this observation suggested the complex function of PUS7 in the TME.

### Analysis of PUS7-related regulatory pathways

**Figure 6:**
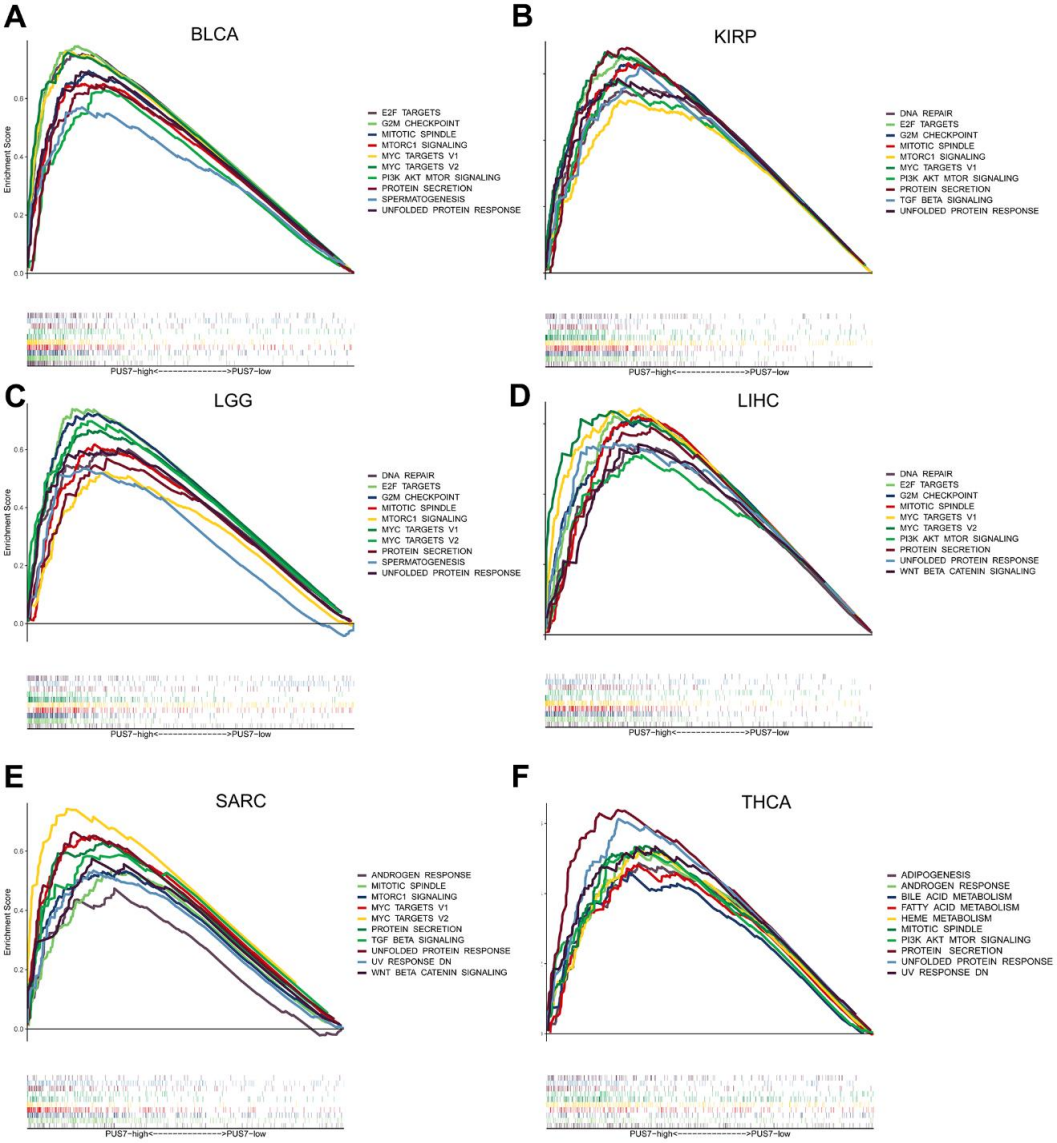
**Gene set enrichment analysis (GSEA) based on the 50 cancer hallmarks.** (**A**–**F**) The GSEA analysis between PUS7-high -low groups in BLCA, KIRP, LGG, LIHC, SARC, and THCA. Each panel on the left and right represents the enriched pathways of the PUS7-high and -low groups, respectively.

**Figure 7:**
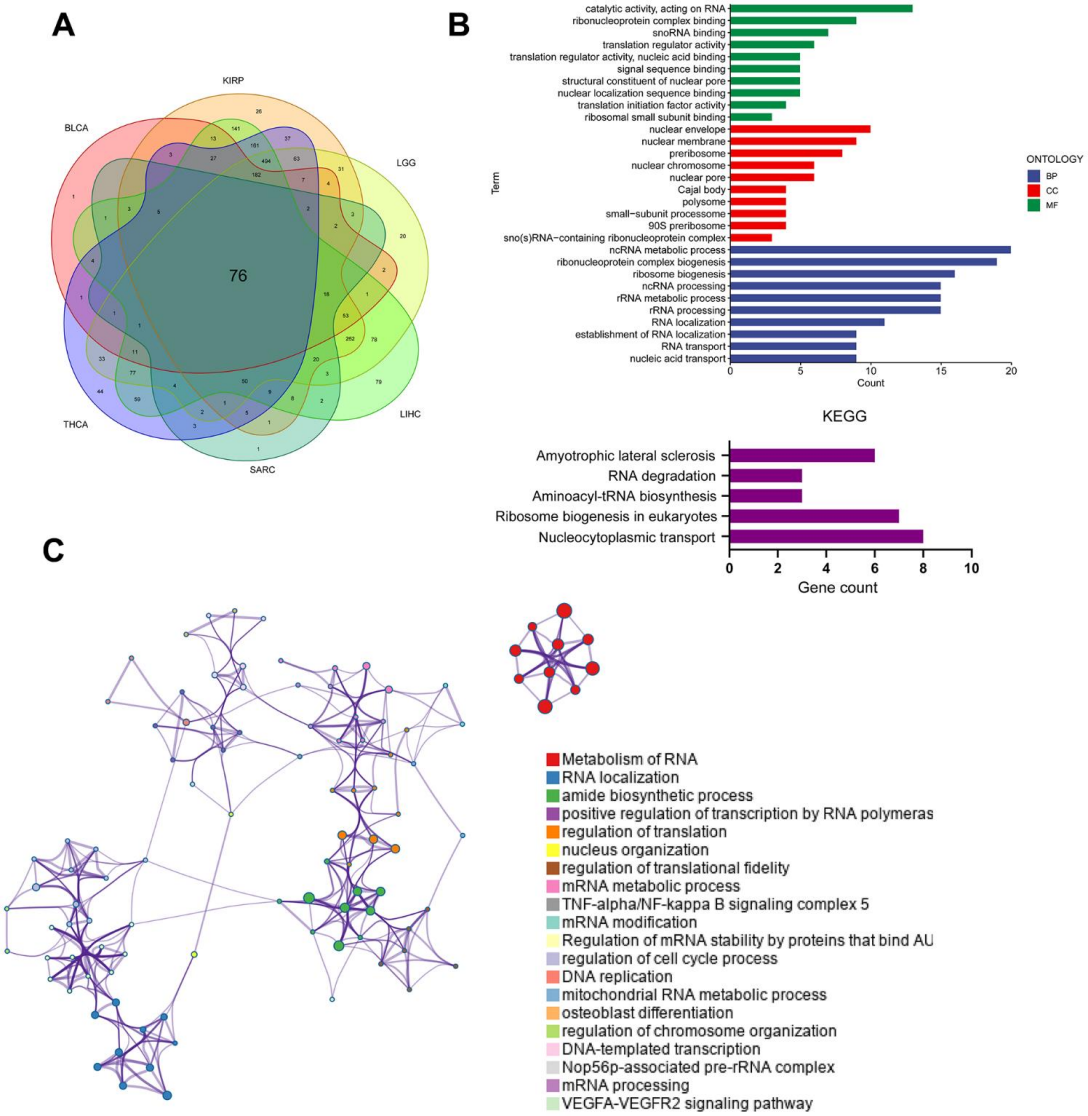
**Analysis of PUS7-related regulatory pathways.** (**A**) The intersecting genes which significantly correlated with DEGs were obtained using Spearman’s correlation analysis (p < 0.05, Cor > 0.4). (**B**) GO (top) and KEGG (bottom) analysis for the PUS7-related regulatory genes. (**C**) PUS7-related regulatory genes were mainly enriched in the mitotic cell cycle and DNA metabolic processes. The interactive network was constructed using the Meta scape online platform.

Cancer patients were divided into the PUS7-high and -low subsets based on PUS7’s median value in six cancer types respectively to explore the oncogenic role of this protein. The gene sets enriched in both groups was identified via gene set enrichment analysis. The top-10 NSE-ranked enriched pathways were visualized in this study. Interestingly, GSEA outcomes showed surprising consistency among the six cancer types, demonstrating the reliability of our findings. Generally, upregulated genes in the PUS7-high subset demonstrated the enrichment of G2M checkpoint, mitotic spindle, PI3K ATK MTOR signaling, and mTORC1 signaling, which were related to cell cycle and proliferation (Figure 6). Moreover, the PUS7-high group exhibited significant enrichment in the DNA repair pathway. The PUS7-related regulatory genes were also investigated in our work. First, patients were classified into PUS7-high and -low subsets based on PUS7’s median values in BLCA, KIRP, LGG, LIHC, SARC, and THCA. Subsequently, differentially expressed gene (DEGs) analysis and Spearman’s correlation analysis were performed in each cancer type, yielding 76 PUS7-related regulatory genes (Figure 7A). The GO analysis identified these regulatory genes were significantly enriched in ncRNA metabolic process, ribosome biogenesis, rRNA metabolic process, RNA location, and complex ribonucleoprotein biogenesis. In addition, these genes were concentrated in nucleocytoplasmic transport and RNA degradation using KEGG analysis (Figure 7B). Finally, the effector function of these regulatory genes was validated using the Metascape online platform (Figure 7C). The results revealed that PUS7-related regulatory genes involved RNA metabolism, RNA location, amide biosynthesis process, nucleus organization, mRNA modification, DNA replication, and osteoblast differentiation.

### PUS7 as a promising biomarker in osteosarcoma

**Figure 8:**
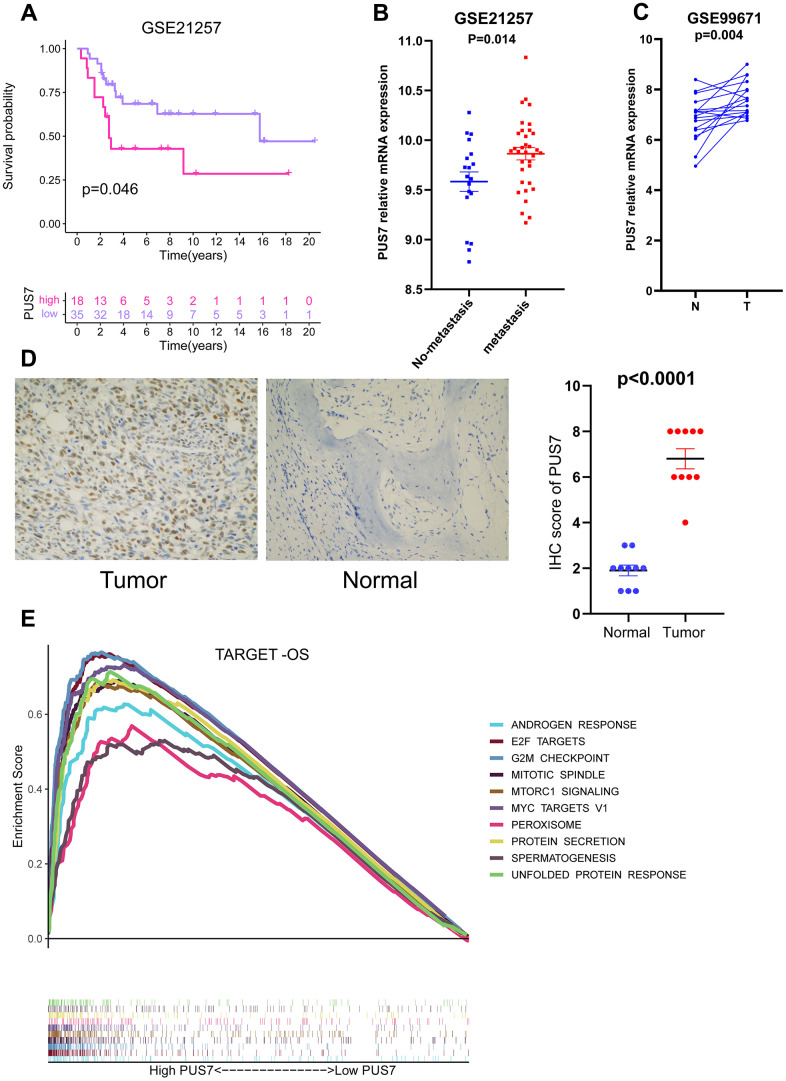
**PUS7 as a promising biomarker in osteosarcoma.** (**A**) Kaplan–Meier analysis of the association between PUS7 expression and OS in GSE21257. (**B**) Comparison of PUS7 expression between non-metastasis and metastasis samples in osteosarcoma. (**C**) Comparison of PUS7 expression between tumor and normal samples in osteosarcoma. (**D**) The expression value of PUS7 in osteosarcoma tissue and adjacent normal specimens determined by IHC analysis. (**E**) The GSEA analysis between PUS7-high and-low groups in the Target-OS cohort.

Our previous analysis confirmed PUS7 expression was markedly elevated in sarcoma tumor tissues. Moreover, PUS7 significantly impacted OS, DSS and DFI in sarcoma patients and was linked to a poor prognosis when highly expressed. Notably, upregulated PUS7 expression was significantly associated with poor outcomes in osteosarcoma. PUS7 may therefore control the growth of osteosarcoma. The PUS7 effector function was also explored using the data from another osteosarcoma study (GSE21257). The Kaplan-Meier survival curve indicated upregulated PUS7 expression was significantly linked to poor outcomes in GSE21257 (Figure 8A). The PUS7 was also significantly upregulated in patients with osteosarcoma metastases, suggesting the modulatory role of this protein in osteosarcoma progression (Figure 8B). The RNA-seq data for osteosarcoma tissue and corresponding paired normal bone tissue was obtained from GSE99671 [20]. PUS7 expression was discovered to be significantly increased in osteosarcoma tissues (Figure 8C). Immunohistochemical results identified that PUS7 was significantly overexpressed in osteosarcoma tissues compared to the corresponding non-cancerous normal ones (Figure 8D and Table 1). Finally, the target-OS cohort cases were categorized into PUS7-high and -low subsets based on PUS7’s median value. The gene set enriched in PUS7-high subset was then determined using GSEA, and the output indicated that G2M checkpoint, mitotic spindle, and mTORC1 signaling were significantly enriched (Figure 8E).

**Table 1:** The basic information on the osteosarcoma patients in this study.

**Patient**	**Gender**	**Age**	**Primary site**	**Admission time**	**Enneking stage**	**Grading**
1	female	16	Distal femur-left	2020.5.16	IIA	II
2	male	10	Distal femur-right	2020.10.12	IIA	II
3	female	12	Distal femur-left	2020.12.25	IIB	II
4	male	16	Proximal humerus-right	2020.10.20	IIIA	III
5	male	8	Distal femur-right	2021.6.22	IIA	II
6	female	14	Distal femur-right	2021.11.14	IIA	II
7	female	12	Proximal tibia-right	2022.4.7	IIA	II
8	male	13	Proximal humerus-right	2022.4.27	IIIB	III
9	male	15	Proximal tibia-right	2022.8.1	IIA	II
10	male	15	Distal tibia-left	2022.6.27	IIB	II

## DISCUSSION

Post-transcriptional gene expression is controlled by a critical mechanism known as RNA modification, which regulates multiple cellular processes, including translation initiation, transcript stabilization, pre-mRNA splicing, and nuclear export promotion [21, 22]. Furthermore, RNA modification links transcription and translation, which are essential for the development of various diseases and determine the fate of cancer cells [23]. The most recently-studied RNA modification and an attractive therapeutic target are M6A, which significantly affects the carcinogenesis and metabolic reorganization of cancer cells [24, 25]. Pseudouridine (ψ) is a C5-glycoside isomer of uridine, which incorporates the C5 atom of the nucleobase into the glycosidic bond [26]. The ψ modifies almost all RNAs, including mRNA, tRNA, and rRNA. In mRNA, ψ incorporation can mediate the conversion of non-sense to sense codons and promote base pairing in ribosomal decoding centers, leading to protein diversity [27]. Furthermore, mRNAs containing ψ in stressed cells exhibited higher stability, suggesting that increased pseudouridylation can enhance cell stability [28]. Recent studies reported that pseudouridylation controls the development of numerous malignancies. For instance, DKC1 binds and stabilizes mRNAs of selected ribosomal proteins based on the pseudouridine synthase activity, thus, promoting colorectal cancer progression in *vitro* and in *vivo* [29]. Furthermore, DKC1 is a trustworthy biomarker for breast and prostate cancers [30, 31]. Therefore, ψ could be a pharmacological target and serve as a biomarker for human cancer. Despite that, PUS7 cancer research is limited to several malignancies, such as glioma, ovarian cancer, and colon cancer. To date, no studies have reported on the commonalities of PUS7 in multiple cancers. Our study confirmed that PUS7 was significantly upregulated in tumor tissues compared to normal ones and accurately predicted the prognosis of cancer patients. In addition, PUS7 was involved in the E2F targets, G2M checkpoint, ribosome biogenesis, and rRNA metabolic process. Most importantly, PUS7 is a promising biomarker for osteosarcoma that possibly regulates osteosarcoma initiation and progression.

The PUS7 mRNA value in 33 different cancer types were first assessed employing TCGA and GTEx data. The results indicated PUS7 mRNA value was significantly upregulated in most cancers except KICH, THCA, and LAML, which exhibited low PUS7 levels. Moreover, the majority of cancer tissues possessed significantly greater PUS7 protein levels than the corresponding paracancerous tissues, based on the HPA database. Previous studies have revealed that PUS7 is upregulated in glioma, ovarian, and colon cancers, which is in line with our results [10, 11, 32]. In conclusion, PUS7 was upregulated in most malignancies and may be considered a diagnostic biomarker. Thus, further in-depth investigations should be performed as PUS7 was highly expressed in various tumor tissues. The PUS7 predictive ability for the cancer patient prognosis was also assessed in this study. It was discovered that PUS7 is a risk factor for 11 cancer types, including ACC, BLCA, LIHC, KIRP, MESO, LGG, KICH, SARC, OS, PAAD, and THCA. High PUS7 expression contributed to the poor prognosis among cancer patients. Our finding suggested that PUS7 is possibly a proto-oncogene. Previous experiments have demonstrated that PUS7 is an unfavorable gene in colon cancer and GBM [11, 13]. Moreover, elevated PUS7 expression was typically linked to advanced TNM across various malignancies, which aligned with the survival analysis outcomes. Therefore, PUS7 is a promising cancer biomarker.

Previous colorectal cancer reported on the PUS7’s regulatory function in PI3K/AKT/mTOR and the Wnt/-catenin signaling pathways [12, 13], which promote tumor cell growth and migration. Furthermore, it has been reported that PUS7 can regulate the metastatic ability of colon cancer cells through the HSP90/PUS7/LASP1 axis [10]. In this study, PUS7-regulated pathways were determined via GSEA. Genes upregulated in the PUS7-high subset displayed enrichment of E2F targets, G2M checkpoints, PI3K ATK MTOR signaling and Mtorc1 signaling, which are connected to cell cycle and proliferation. For instance, PI3K-Akt-mTOR is a crucial kinase that controls and activates essential cellular processes, including proliferation, transcription, translation, survival, and growth [33]. In pathological circumstances like cancer, the PI3K-Akt-mTOR signaling pathway is essential for cell survival and proliferation and regulates autophagy and apoptosis process [34]. The GSEA results from this study displayed remarkable consistency among the six cancer types, indicating the reliability of the study output. A total of 78 PUS7-related regulatory genes were explored in this study. The functional analysis revealed these regulatory genes exhibit significant enrichment in the ncRNA metabolic process, rRNA metabolic process, RNA location, and complex ribonucleoprotein biogenesis. Furthermore, these genes regulated RNA metabolism and location, amide biosynthesis, nucleus organization, mRNA modification, DNA replication, and osteoblast differentiation. In conclusion, PUS7 plays a role in ribosome biogenesis, which is vital for cell proliferation, differentiation, apoptosis, development, and transformation [35]. Additionally, Prakash et al. discovered that metastasis of cancer cells could be promoted by synthesizing neo-ribosomes [36].

Cui et al. discovered PUS7 regulates GSC development and carcinogenesis by modifying TYK2 translation via PUS7-dependent tRNA pseudouridylation [11]. Our study emphasizes that PUS7 regulates rRNA metabolic process and ribosome biogenesis. Ribosomes are made up of rRNA and proteins, which is crucial hub for protein synthesis. Tumor growth requires elevated ribosome biogenesis. Targeting ribosomes is an important strategy for cancer therapy [37]. PUS7 probably controls rRNA metabolism to encourage the cancer growth. Previous studies have indicated that deletion of PUS7 in *Candida albicans* results in defective rRNA processing and reduced cell surface hydrophobicity [38]. The precise mechanisms by which PUS7 controls rRNA metabolism in cancer have not been studied. Increased attention to this field is necessary.

The TME is the cellular setting in which cancer cells exist, comprising non-cancerous cells, their components, and the molecules they produce and secrete. The TME determines the clinical outcome of malignancies, drug resistance, and immune evasion [19]. Cancer therapy can now be achieved by manipulating different cell types in TME, and some of these methods are now applied in clinic [39]. Thus, it is essential to explore the effector function of oncogenes in the TME to develop effective cancer therapeutic approaches. One of the best tools to study the TME is by using scRNA-seq technology. This study found that PUS7 was expressed in malignant cells, as well as in endothelial, fibroblast, and immune cells, including macrophages and T cells. Likewise, we focused on PUS7’s expression in osteosarcoma cell types and discovered that myeloid cells, fibroblasts, and cancer cells had the highest PUS7 expression. In summary, further study is required due to the complexity role of PUS7 in the TME.

Sarcoma is a rare malignant tumor that originates from mesenchymal tissue. It can be classified as soft tissue sarcoma (STS) and bone sarcoma (BS). Most SARCs have a high rate of recurrence or metastasis following local surgery and are unresponsive to radiation or chemotherapy. Several pre-clinical studies on immunotherapy for sarcoma patients have yielded positive responses [40]. Bioinformatics analysis in sarcomas is less popular than in other tumors. Sarcoma biomarker exploration has also been unsatisfactory. The PUS7 expression was significantly upregulated in sarcoma tumor tissues. Moreover, PUS7 significantly impacted the OS, DSS, and DFI in sarcoma patients. Thus, PUS7 potentially controls sarcoma progression. Osteosarcoma is a type of sarcoma, and high PUS7 expression was connected with shorter OS in osteosarcoma patients. Hence, we further analyzed the role of PUS7 in osteosarcoma. PUS7 expression was significantly upregulated in osteosarcoma tissues and was linked to worse prognoses in osteosarcoma patients. Additionally, PUS7 was significantly upregulated in patients with osteosarcoma metastases, indicating the regulatory role of PUS7 in the tumor progression. Therefore, PUS7 is a reliable biomarker and a potential therapeutic target for osteosarcoma. Our current study has several limitations. Despite the extensive sequencing data used in this study (33 cancer types and approximately 10,000 patients), there were few osteosarcoma sequencing datasets. Thus, future studies should include more clinical cohorts to improve the accuracy of the findings.

In summary, PUS7 is a putative pan-cancer biomarker that can reliably forecasts cancer patients’ prognosis, including ACC, BLCA, LIHC, KIRP, MESO, LGG, KICH, SARC, OS, PAAD, and THCA. In addition, the bioinformatics output indicated the regulatory role of PUS7 in cell division and cycle, ribosome biogenesis, and the rRNA metabolic process. Most importantly, PUS7 may control osteosarcoma initiation and progression.
